# Effects of Lipid Saturation on the Surface Properties of Human Meibum Films

**DOI:** 10.3390/ijms19082209

**Published:** 2018-07-28

**Authors:** Yana Nencheva, Aparna Ramasubramanian, Petar Eftimov, Norihiko Yokoi, Douglas Borchman, Georgi As. Georgiev

**Affiliations:** 1 Department of Optics and Spectroscopy, Faculty of Physics, St. Kliment Ohridski University of Sofia, Sofia 1164, Bulgaria; yana.dim.nen@gmail.com; 2 Department of Ophthalmology and Visual Sciences, University of Louisville, Louisville, KY 40292, USA; aparna.ramasubramanian@louisville.edu (A.R.); douglas.borchman@louisville.edu (D.B.); 3 Department of Cytology, Histology and Embryology, Faculty of Biology, St. Kliment Ohridski University of Sofia, Sofia 1164, Bulgaria; petareftimov@abv.bg; 4 Department of Ophthalmology, Kyoto Prefectural University of Medicine, 602-8566 Kyoto, Japan; nyokoi@koto.kpu-m.ac.jp

**Keywords:** human meibum, acyl chain saturation, Langmuir films, tear film stability, surface properties

## Abstract

Elevated levels of acyl chain saturation of meibomian lipids are associated with vastly different effects: from enhanced tear film (TF) stability in infants to shortened TF breakup time in meibomian gland disease patients. Thus it is important to study the effect of saturation on the surface properties of human meibum (MGS). Therefore, MGS films (1, 2, 3, 4, 5, 10, 25, 50, 67, and 100% saturation) were spread at the air/water interface of a Langmuir surface balance. The layers’ capability to reorganize during dynamic area changes was accessed via the surface pressure (π)-area (A) compression isotherms and step/relaxation dilatational rheology studies. Film structure was monitored with Brewster angle microscopy. The raise in the % (at ≥10%) of saturation resulted in the formation of stiffer, thicker, and more elastic films at π ≥ 12 mN/m with the effects being proportional to the saturation level. At the same time, at low (≤10 mN/m) π the raise in saturation resulted in altered spreading and heterogeneous structure of MGS layers. The strong impact of saturation on MGS surface properties correlates with our recent spectroscopy study, which demonstrated that saturation induced increase of MGS acyl chain order, phase transition temperature, and cooperativity.

## 1. Introduction

Human meibomian gland secretion (MGS), also termed meibum, is a composite lipid-rich mixture consisting of >90% nonpolar lipids (primarily wax- and sterol-esters and triacylglycerols) and <10% polar amphiphilic lipids, ((*O*-acyl)-ω-hydroxy fatty acids (OAHFA), and some phospholipids) [1,2,3,4]. As it is the major constituent of the tear film lipid layer (TFLL) covering the air/tear surface, MGS properties are supposed to play crucial role for tear film (TF) stability in health and in dry eye disease, which affects the quality of life and productivity of 10–30% of human population worldwide [5,6]. Currently, meibomian gland dysfunction (MGD) resulting in quantitative and qualitative alterations of MGS is considered as the world-leading cause of dry eye syndrome (DES), with up to 86% of all DES patients showing signs of MGD [7]. A considerable effort has been made to study the structure and properties of MGS films at the air/water interface in vitro as they may provide relevant information about the TFLL performance at the tear surface in vivo. MGS was found to form a thick viscoelastic duplex film composed of (i) a monomolecular layer of amphiphilic polar lipids at the aqueous surface and (ii) an unstructured lipophilic suspension of lipid lamellar–crystallite particulates immersed in a continuous liquid phase, located on top and facing the air [8,9,10,11]. Based on the expertise of thin film research, it is thought that even minor changes of the MGS layer composition may have major impact on its structure and surface properties if the lipid-spreading or film-packing density is altered [12].

The presence of double bonds (unsaturation) in the hydrocarbon chains of lipids affects the conformational order (fluidity) of meibum [13,14,15,16,17,18,19] and its acyl chain-melting phase transition temperature [18,19,20], which is supposed to have major physiological impact. The TF of infants, with a noninvasive breakup time (NIBUT) of 32.5 ± 5.2 s [21,22] is more saturated than that of adults with a less stable TF (NIBUT = 15.73 ± 8.89 s) [23,24]. It is interesting that, although hydrocarbon chain saturation does not change with aqueous-deficient dry eye [25], it may increase with MGD [26], i.e., a condition associated with decreased TF stability [1,7,27,28,29]. The lipid phase transition temperature and order of meibum from donors with MGD are higher compared to normal age-matched donors [17,19,30]. Apart from lipids, MGS may contain up to 22 wt % nonlipid components (proteins, salts, polysaccharides) [31] that may vastly differ, both quantitatively and qualitatively, between infant MGS and MGD meibum. If MGD samples were found to be rich in insoluble keratinous particulates [32], MGS from infants contains a high amount of lactoferrin and serum albumin [33].

Thus it is important to study the effect of varying effects of (un)saturation on the properties of human meibum. In our previous study [16], MGS from an adult person was subjected to catalytic hydrogenation obtaining various degree of acyl chain saturation: 0% (intact meibum), 1, 2, 3, 4, 5, 10, 25, 50, 67, and 100% (corresponding to total saturation and complete absence of double bonds in the acyl chains). Nuclear magnetic resonance (NMR) and Fourier-transform infrared (FTIR) spectroscopy measurements demonstrated that hydrocarbon chain saturation increased lipid order and the phase transition temperature of the samples and was directly related to changes in cooperativity, enthalpy, and entropy. It was supposed that acyl chain saturation may account for the alteration in lipid phase transition parameters and the decrease of TF stability observed with age.

As the ultimate role of meibum in vivo is to spread on the tear surface and to contribute towards optimal functionality of TFLL, as a next step, the performance of these intact (0% saturation) and catalytically-saturated (1, 2, 3, 4, 5, 10, 25, 50, 67 and 100%) MGS samples on the air/water surface is examined with Langmuir surface balance. Langmuir surface balance studies provide insights into factors that influence lipid film spreading and structure on an aqueous surface [8]. For instance, this approach showed that temperature [34], proteins [35], squalene [36], sebum [37], and drugs [38] influence the rheology of meibomian layers. The films’ capability to reorganize during dynamic area changes was evaluated through surface pressure-area compression isotherms. The layers’ dilatational rheological properties were probed via the step/relaxation method through Fourier analysis (in the 1–10^−5^ Hz range) and by exponential decay modeling of the relaxation transients [8,39,40]. This approach allows evaluating the capability of surface films to store energy and to recover their structure when subjected to rapid deformation as the ones on the ocular surface. The films’ structure was monitored with Brewster angle microscopy.

## 2. Results

### 2.1. Surface Pressure-Area Isotherms

**Figure 1:**
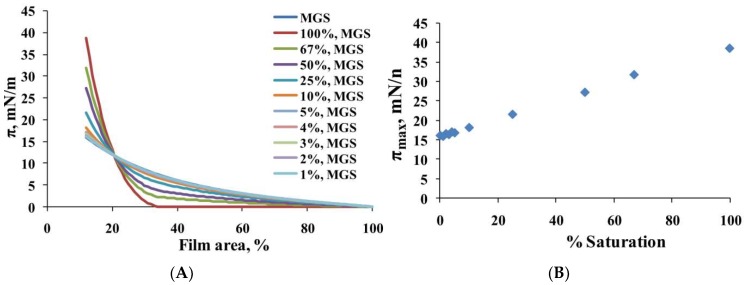
(**A**): Surface pressure (π)/area isotherms of human meibum (MGS) films, intact and with different degrees of catalytic saturation; (**B**): The dependence of maximum surface pressure (π_max_) achieved at minimal surface area (at the completion of compression) on the degree of acyl chain catalytic saturation in MGS films. The higher the molecular packing density at the interface is, the higher π_max_ is; higher cohesion between the lipid tails is thought to strengthen molecular packing (see Section 3 for details).

The analysis of the surface pressure (π)/area (A) isotherms of MGS films (Figure 1, panel A) showed that the catalytic saturation (at ≥10% saturation) of meibum acyl chains resulted in: (i) a decrease of the lift-off area (the area at which surface pressure raises from zero) from 95% of the initial film area (intact MGS) to 35% of the initial area (at 100% saturation) and (ii) an increase of the maximum surface pressure (π_max_) achieved at minimal surface area at the completion of film compression. As shown at panel B of Figure 1, the value of π_max_ was 17mN/m for intact MGS (0% saturation) and, at ≥10% acyl chain saturation, π_max_ gradually rose with the level of catalytic saturation to reach 38 mN/m for 100% saturated MGS.

**Figure 2:**
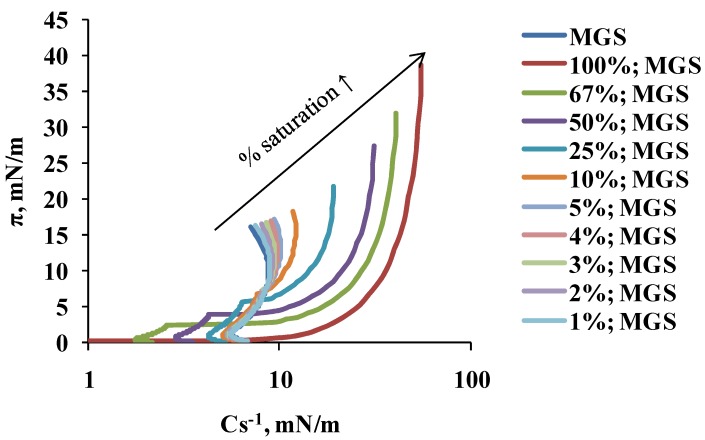
The dependence of surface pressure on isotherm reciprocal compressibility (Cs^−1^; shown on log scale) of MGS films (intact and with different degree of catalytic saturation). Cs^−1^ was calculated from the π/A isotherms shown at the upper panel of Figure 1 by Equation (2) (see Section 4.2.1).

The acyl chain saturation-induced changes in MGS material properties were well manifested in the dependence of the reciprocal compressibility modulus, Cs^−1^ (calculated from the π/A isotherms via Equation (2), see Section 4.2.1), on the % of saturation (Figure 2). It can be seen that the increase of % saturation raised the maximum value of Cs^-1^ from 8–10 mN/m (at 0–5% saturation of MGS) to 55 mN/m (at 100% saturated MGS), thus suggesting the formation of stiffer films with the raise of acyl chain saturation (at ≥10% saturation).

The lack of effect of ≤5% saturation on surface pressure/area isotherms and its proportionally increasing impact at ≥10% saturation on π_max_ and Cs^−1^ correlate with the similar effects of % of saturation on acyl chain order, phase transition temperature, and cooperativity found in our previous study [16].

**Figure 3:**
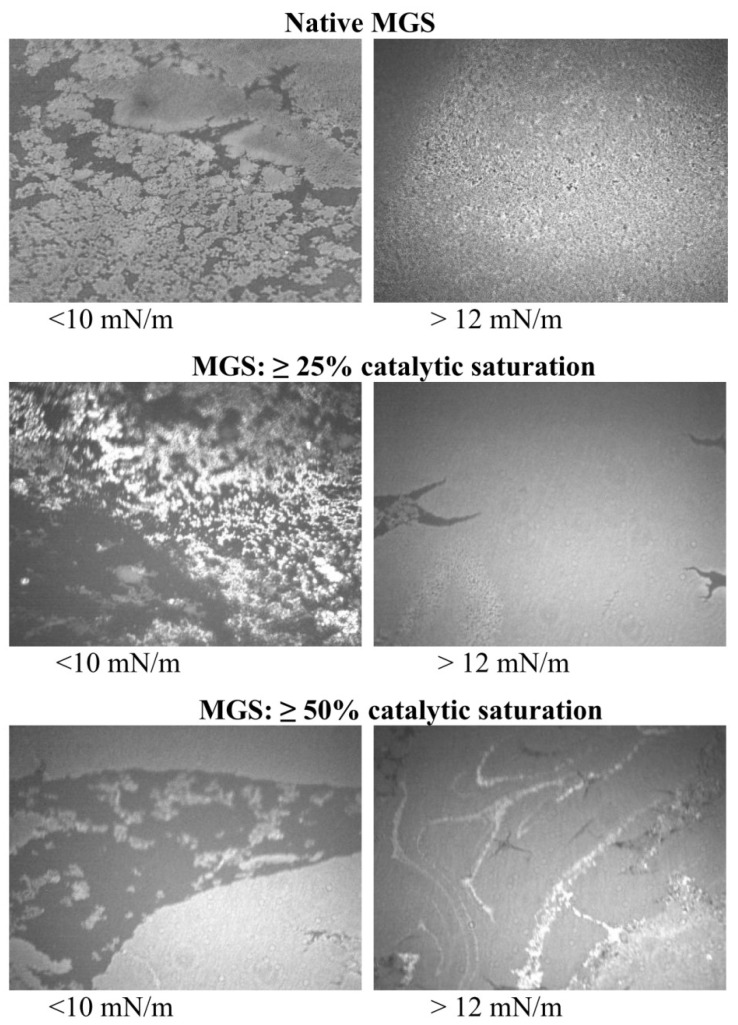
Brewster angle microscopy (BAM) micrographs (3000 µm × 3000 µm; 40× magnification) of MGS films with different degrees of catalytic saturation. The intensity of the right panel images is intentionally attenuated for better visual perception.

As reported in multiple studies [8,12,38,39], Brewster angle microscopy images (Figure 3) showed that, at the lift-off surface pressure MGS samples already formed heterogeneous films consisting of thin monolayer regions (dark areas) and thick aggregates of multilayer thickness (bright areas) at low (≤10 mN/m) surface pressures. With the increase of π at further compression (usually starting from the π values corresponding to inflexion points of the π/Cs^−1^ dependencies [8,38,39]), the thick regions enclosed to form a rough and continuous multilayer, while the thin monolayer regions almost disappeared. As can be seen at a low % of acyl chain saturation, the multilayer aggregates appeared rougher and more uniformly distributed at the air/water interface (especially at lower π values) compared to MGS at >50% saturation. For these highly saturated samples at low surface pressure, the film consisted of larger dark regions and bright thick meibum islands whose uniform appearance indicates tight molecular packing characteristic for gel-like structures [41]. At further compression, these bright islands approached and enclosed together.

### 2.2. Dilatational Rheology

**Figure 4:**
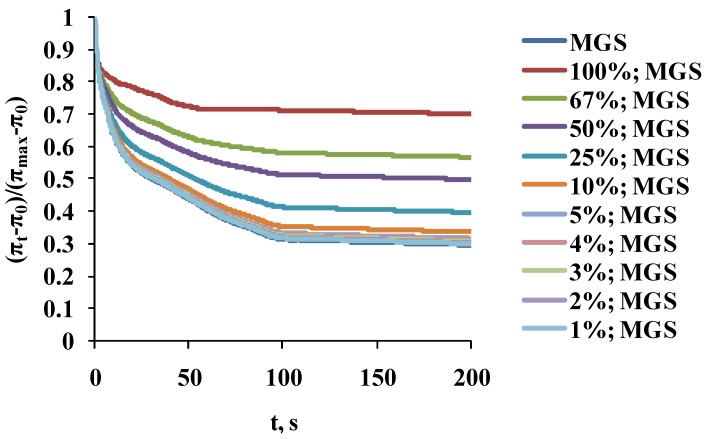
Surface pressure stress-relaxation transients of MGS films, intact and with different degree of catalytic saturation.

The stress relaxation transients (Figure 4) of samples with 1–10% saturation were almost identical (as shown by *p* ≥ 0.1 obtained by ANOVA comparison between the curves [42]) to the one of native MGS (0% saturation). For the rest of the layers, the increase in catalytic saturation shifted the surface pressure relaxation transients to higher π increment values (a manifestation of increased elasticity) and changed the shape of the transients (i.e., there was change in the structure and molecular rearrangement processes in the films).

**Figure 5:**
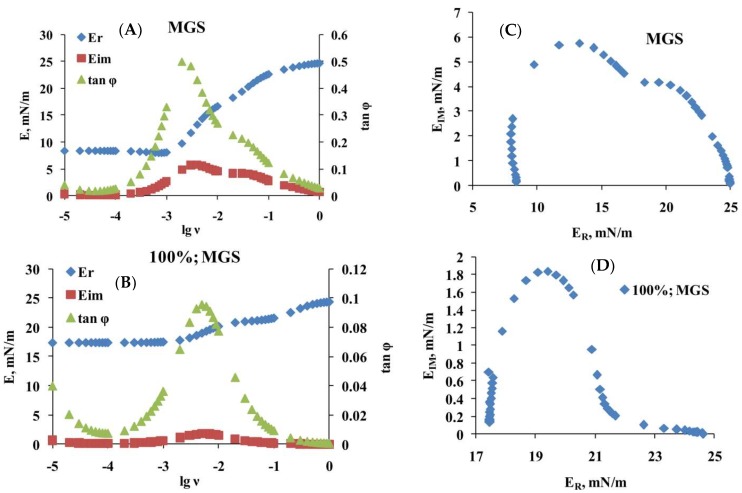
Rheological parameters obtained via Fourier transformation (**A**,**B**) and Cole-Cole (E_R_ vs. E_IM_) plots (**C**,**D**) of intact and 100% saturated MGS films, respectively. The results for 25, 50, and 67% saturated MGS are presented as a Supplementary file.

The relaxation curves for intact MGS and for meibum with 25, 50, 67, and 100% acyl chain saturation were subjected (Equations (3) and (4) in Section 4.2.2) to Fourier transformation analysis [8,39,40], which showed (left panels of Figure 5 and the information in the Supplementary file) that, with the increase of saturation, there was rise in the value of the elastic part, E_R_, of the complex modulus, particularly at low, ≤10^−3^ Hz, frequencies and that the value of tan φ (i.e., the ratio between E_IM_ and E_R_) decreased particularly in the frequency region of 10^−3^ to 10^−1^ Hz.

The plots shown at panels A and B of Figure 5 were subjected to further analysis by constructing (panels C and D of Figure 5 and the information in the figure S1) their corresponding Cole–Cole plots (i.e., graph of E_IM_ vs. E_R_). 

**Figure 6:**
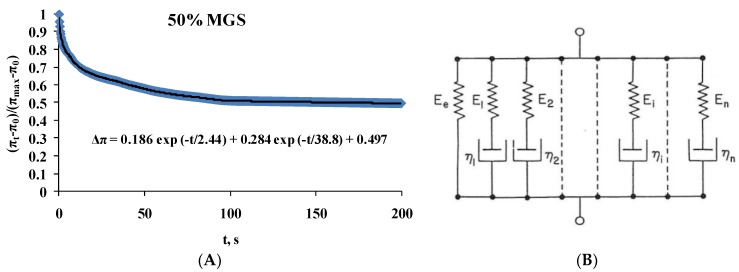
(**A**) Example of double exponential decay fit (R^2^ = 0.98) with Equation (1) of the stress relaxation transients of 50% saturated MGS film. The values of the fitted parameters are presented on the chart; (**B**) Schematic presentation of the generalized Maxwell model (represented by Equation (1)) consisting of parallel Maxwell spring-and-dashpot elements and a spring element denoting the equilibrium elasticity E_e_. See main text for details.

It can be clearly seen that two peaks were observed in the Cole–Cole plots, at E_R_ of 12 and 20 mN/m for intact MGS [43,44]. The peak at 20 mN/m became less pronounced with the increase of acyl chain saturation. This behavior suggests that relaxations are governed by two processes (one of which becomes less pronounced with the rise in % saturation) and thus the raw transients (Figure 4) can be fitted (Figure 6A) with a double exponential decay equation [43,44,45,46]:

Δπ = A_1_ exp (−t/τ_1_) + A_2_ exp (−t/τ_2_) + Δπ_EQ_. (1)

Here the number of exponents corresponds to the number of relaxation processes: A—pre-exponential factor reflecting the contribution of the individual term to the relaxation; τ—characteristic relaxation time; Δπ_EQ_—plateau value.

In the framework of the generalized Maxwell model (panel B of Figure 6), the equation represents a rheological system of parallel elements in which each exponential term corresponds to a Maxwell spring-and-dashpot element and Δπ_EQ_ to a spring element denoting equilibrium elasticity [46,47]. The relaxation time of each Maxwell element is defined as the ratio between its viscosity and elastic modulus (τ_i_ = η_i_/E_i_).

The output of applying Equation (1) is summarized at Table 1.

As can be seen, two characteristic relaxation time regimes were observed, fast (τ < 0.8–3.3 s) and slow (τ = 32.7–42.6 s), with the pre-exponential factor of the “slow” exponent decreasing with the increase of acyl chain saturation. The Δπ_EQ_ value also increased, which reflects the rise of the plateau value of the surface pressure increment of the relaxation curves.

## 3. Discussion

Elevated levels of acyl chain saturation of meibomian lipids occur both in infants and in MGD patients, and are associated with vastly different effects: from enhanced TF stability (in babies) to short NIBUT of TF (in people with MGD) [23,24,27,28,29]. The spontaneous blink rate of adults is approximately 20 blinks per minute, much higher than that of infants, which blink less than once a minute [21,22,23,24]. The spontaneous blink rate is, in turn, related to the TF break-up time. NIBUT is as high as 35 s in infants and decreases to 8–16 s in adults. TF break-up time is even lower (5 s) in adults with MGD [7,25,26,27]. Thus it is important to see what alterations occur in the surface properties of human meibum films with the rise of catalytic saturation.

The current study shows that the rise in the % of acyl chain saturation results in formation of stiffer, thicker, and more elastic films at high (π ≥ 12 mN/m) surface pressures with the effects being proportional to the level of saturation. It was found that, in order for the effects to become significant, acyl chain saturation should be ≥10%. These findings agree very well with our recent spectroscopic study, which demonstrated a similar impact of the % of saturation on acyl chain order, phase transition temperature, and cooperativity [16]. These findings align very well with the tighter molecular packing and stronger cohesion between saturated lipid tails compared to the sparser molecular packing imposed by the steric interactions in double bond-containing moieties [48,49,50]. The tighter molecular packing is, in turn, supposed to result in enhanced elasticity of lipid monolayer and bilayer membranes [49,50]. The results also correlate very well with the supposed roles of TFLL in vivo, i.e., to enhance the elasticity and mechanical stability of the air/tear surface and to serve as a barrier to evaporation of the underlying aqueous tear fluid [8,12].

At the same time, the increased cohesion between the acyl chains poses certain limitations as well. Namely, with the increase of lipid tail saturation, the spreading of MGS films was impaired, as manifested by (i) the increase in the lift off area, (ii) the shift of the saturated films’ isotherms to lower π values at ≥20% of the initial film area, and (iii) the heterogeneous structure of the meibomian layers (containing large dark regions separating the thick bright islands) at π ≤ 10 mN/m. Such observations align with the supposed qualitative deficiencies of MGD meibum [8,14,15,16,17,18], namely abnormally high melting temperature resulting in high bulk viscosity, which in physiological conditions hampers the expression of MGS from the orifices of the meibomian gland ducts and also suppresses the spreading of meibum over the aqueous tear layer at the ocular surface.

Thus, in order for the potential beneficial effects of acyl chain saturation to be harnessed, it is necessary the spreading of saturated MGS to be enhanced. In vivo at the ocular surface this can be done in two ways—via amphiphilic polar lipids and by tear proteins. Indeed, although the data on the type and amount of polar lipids in tears are still controversial, it is reported by independent groups that the levels of (*O*-acyl) ω-hydroxy fatty acids (OAHFA), cholesteryl sulfate, and eventually phospholipids are significantly decreased in MGD [1,4]. The protein content between infants, normal adults, and MGD patients is also vastly different. In infants, it is known that MGS has a high content of lactoferrin and serum albumin, both known to possess surface activity and good miscibility with lipids [28,33]. Both MGS and aqueous tears in infants and healthy adults also contain lacritin, a multifunctional protein that was found to enhance MGS surfactant properties. It is the only protein found to be downregulated in dry eyes, with its concentration respectively being grossly diminished [1,28]. In contrast, hot stage cross-polarized light microscopy revealed that meibum collected from patients with MGD showed an increased presence of nonlipid, nonmelting, nonbirefringent, chloroform-insoluble inclusions of a protein nature (positively stained for cytokeratins) that altered MGS film-melting characteristics and disrupted the structural integrity of TFLL and its proper functionality at the air/tear interface [32]. This finding is in line with the previously reported linear correlation between protein content and meibum-melting temperature, which, in turn, is expected to hamper the expression and spreading of MGD meibum [26].

Thus, it can be concluded that the degree of acyl chain saturation will have significant influence on MGS and TFLL performance at the ocular surface in vivo. Whether its impact will be positive (enhancement of TFLL viscoelasticity and evaporative barrier functionality) or negative (impaired spreading) will depend on the availability of polar lipids and certain proteins in TF and on the interactions of MGS with these compounds [1,4,12].

The results have certain implications to pharmaceutical design as well. Ophthalmic formulations like eyedrops and topical (nano)emulsions may supplement the TFLL by range of oils that are generally recognized as safe and efficient [1,12,39]. Usually, little attention is paid to the fact that these oils significantly differ in their degree of acyl chain unsaturation and structure. Mineral oils consist of saturated hydrocarbons (alkanes) and coconut oil is enriched (⁓90%) with saturated fats as well. In contrast, castor oil and, in particular, sesame oil are rich in a variety of mono- and poly-unsaturated fatty acids. Thus, oils alone, or in combination with amphiphilic lipids, may provide a tool to fine tune the balance between the spreading capacity and the viscoelastic and evaporation barrier properties of TFLL in vivo [1,12,16].

## 4. Materials and Methods

### 4.1. Materials

Meibum lipid was expressed from the eyelids of healthy volunteers with no signs of dry eye and was collected with a platinum spatula as previously described [51]. The expressed meibum was dissolved in 1.5 mL CDCl_3_. Half the pooled meibum was decanted to be catalytically hydrogenated. Saturated meibum was prepared as in our previous study [16]. Platinum (IV) oxide (7.4 mg) was used as a catalyst to reduce the samples with hydrogen at room temperature and atmospheric pressure for ~4 h with stirring. Then the catalyst was separated from the solution by centrifugation. The level of saturation was confirmed by H-NMR analysis, as described in detail in Sreshta et al., 2017 [16]. The catalytically-saturated samples were quantitatively mixed with a sample that was not catalytically saturated to provide mixtures containing 1%, 2%, 3%, 4%, 5%, 10%, 25%, 50%, and 67% of catalytically-saturated meibum.

### 4.2. Langmuir Trough Studies

#### 4.2.1. Compression Isotherms

Surface pressure–area (π-A) isotherms were measured [8,36,52] using Langmuir surface balance µ Trough XS, area 135 cm^2^, volume 100 mL (Kibron, Helsinki, Finland) by the Wilhelmy wire probe method (instrumental accuracy 0.01 mN/m). The trough subphase was a physiological saline solution buffer (PBS, pH 7.4). Human MGS (with different levels of catalytic saturation), dissolved in chloroform, was deposited (35 µL of 1 mg/mL) over the air/saline surface with a microsyringe (Hamilton Co., Reno, NV, USA). The trough was positioned under an acrylic cover to protect the surface from dust and to suppress the evaporation of the saline solution subphase. After 15 min were given for chloroform evaporation, film compression was performed by two symmetrically-moving barriers. Dynamic compression–expansion isocycling of the layer area was done with the maximum barrier’s rate (70 mm/min), at which there was no film leakage. Ten consecutive cycles were performed with each film studied. Normally after the third cycle, the shape of the π(A) curves remained constant and those π(A) isotherms were presented and analyzed. All isotherms were repeated at least three times; the difference between the repetitions was less than 2%. The experiments were done at 35 °C, i.e., the physiological temperature of the ocular surface. The films’ morphology was monitored by Brewster Angle Microscopy (MicroBAM, KSV-NIMA).

Film surface compressional modulus, Cs^−1^, at a given surface pressure was calculated from π/A compression isotherms using the equation [53]:

C_s_^−1^ = A_π_ (dπ/dA)_T_ (2)

where Aπ is the area at the indicated π. The inflexion points in the π/Cs^−^^1^ dependencies indicate the surface pressures at which significant reorganization of the surface film takes place in the course of the film compression.

#### 4.2.2. Stress-Relaxation Studies via the Small Deformations Method

In order to gather information about the dilatational viscoelasticity of meibum films, intact and catalytically saturated, the relaxation of the surface pressure was monitored after a small rapid compression deformation was applied to the surface film. Firstly the film was compressed to initial surface pressure, π_0_, of 15 mN/m. Then, the lipid film was instantaneously and slightly contracted with a compression step, ∆A/A_o_ = 5 ± 1% (A_o_ is initial film area, and ∆A—area change). As discussed elsewhere [40,54,55] no assumptions are made about the surface film structure or the physical nature of the relaxation processes (e.g., diffusion to/from the bulk solution, molecular rearrangements, exchange with secondary adsorption layers, etc.). The relaxation transient is presented in normalized coordinates (π_t_ − π_0_)/(π_max_ − π_0_) = f(t), where π_t_ is the momental value of the surface pressure and π_max_ is its maximal value at the start of the relaxation. The dependence of the real, E_R_, and imaginary part, E_IM_, of the complex dilatational elasticity modulus E*(ν) on frequency, ν, can be obtained via Fourier transformation, F, of the relaxation transients [8,55,56]:

(3) $$E^{*}\left(\nu \right)=E_{R}\left(\nu \right)+iE_{IM}\left(\upsilon \right)=\frac{F\left\{d\Delta \pi \left(t\right)/dt\right\}}{F\left\{dlnA/dt\right\}}=\frac{i6.28\nu }{\Delta A/A_{0}}\overset{\infty }{\underset{0}{\int }}\Delta \pi \left(t\right)exp\left(-i6.28\nu t\right)dt$$

Here E_R_ accounts for the elasticity of the surface film, while E_IM_ set by the product of ν η_d_ (η_d_ is the dilatational viscosity) accounts for the dissipative, viscous properties of the film. The number 6.28 is a brief denotement of the doubled Archimedes constant (2 × 3.14159…). The Fourier analysis of the relaxation transients was performed as previously described [54,55,56] utilizing commercial Fourier transform software provided by Kibron Inc. (Helsinki, Finland).

After the real and imaginary parts of the complex modulus were calculated, the tangent of phase angle was computed:

tan*φ* = E_IM_/E_R_ (4)

If E_R_ > E_IM_, then tan *φ* < 1 and the film is predominantly elastic. On the contrary, if E_R_ < E_IM_, then tan *φ* > 1 and the film is predominantly viscous.

## Figures and Tables

**Table 1:** Maxwell rheological model equations for MGS film samples. All the equations fit the raw transient at Figure 4 with R^2^ ≥ 0.98.

Composition	Maxwell Rheological Model Equation
MGS	Δπ = 0.267exp(−t/3.3) + 0.42exp(−t/42.6) + 0.29
100%; MGS	Δπ = 0.144exp(−t/0.8) + 0.145exp(−t/32.7) + 0.699
67%; MGS	Δπ = 0.17exp(−t/2.5) + 0.229exp(−t/42.5) + 0.56
50%; MGS	Δπ = 0.186exp(−t/2.4) + 0.284exp(−t/38.5) + 0.497
25%; MGS	Δπ = 0.22exp(−t/2.8) + 0.353exp(−t/39.8) + 0.396

## Supplementary Materials

Supplementary materials can be found at http://www.mdpi.com/1422-0067/19/8/2209/s1.

## Abbreviations

TF	Tear film
TFLL	Tear film lipid layer
MGS	Meibomian gland secretion (or simply meibum)
MGD	Meibomian gland disease
π	Surface pressure
BAM	Brewster angle microscopy
