# Supplementary material for: Effects of Lipid Saturation on the Surface Properties of Human Meibum Films
Source: Int J Mol Sci. 2018 Jul 28;19(8):2209. doi: 10.3390/ijms19082209 (PMC6121396; doi:10.3390/ijms19082209)

Supplementary file: Fourier transformation analysis and Cole-Cole plots of human meibum films with 25-67% of catalytic saturation of the lipid acyl chains

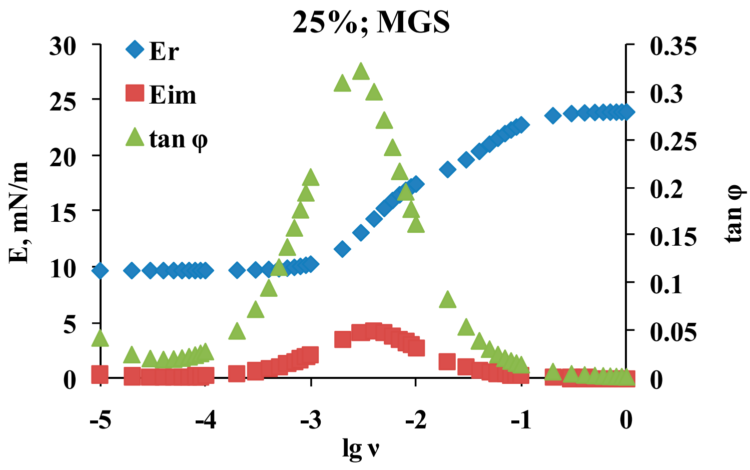

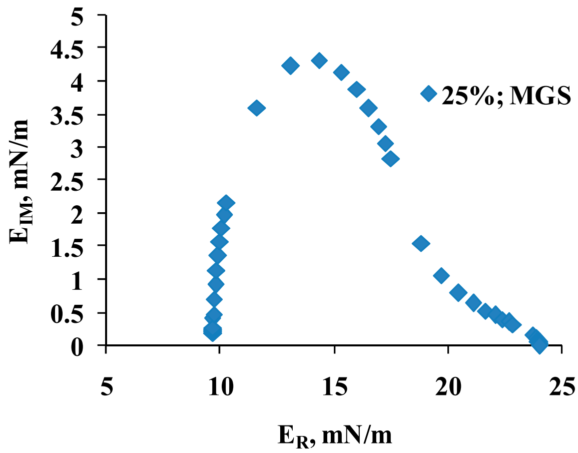

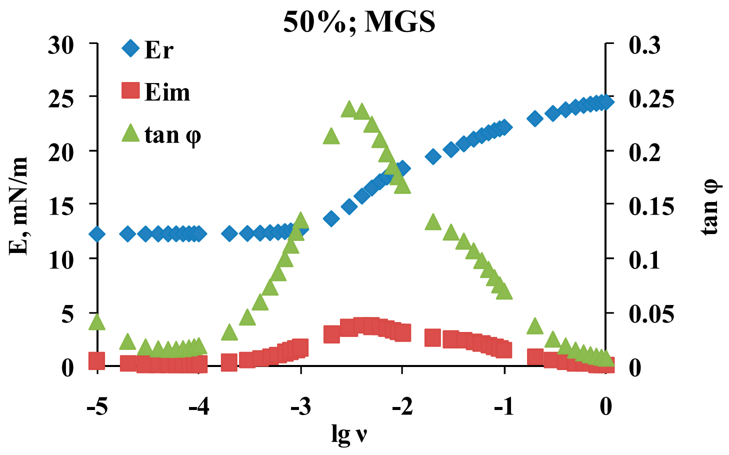

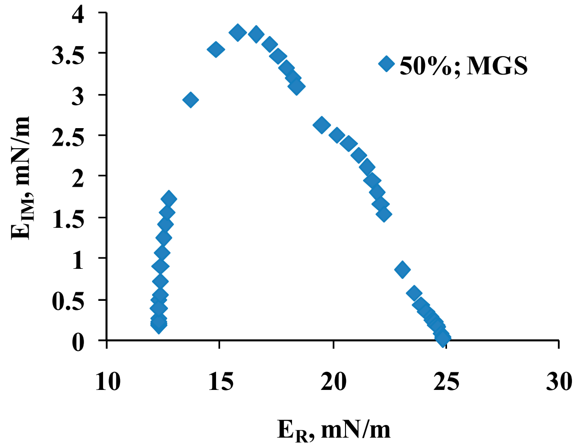

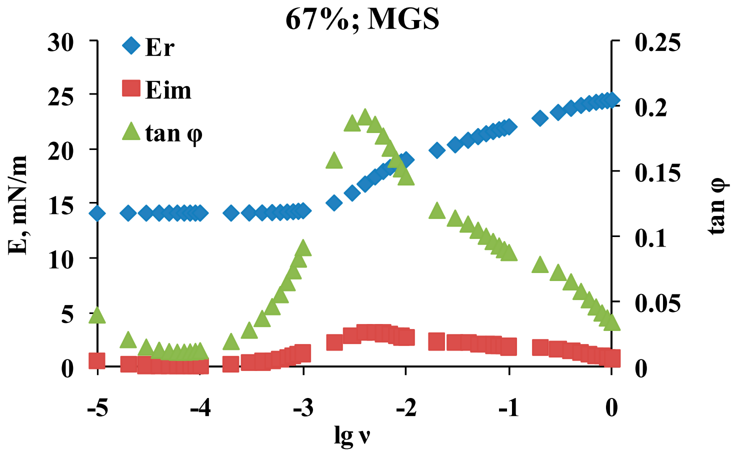

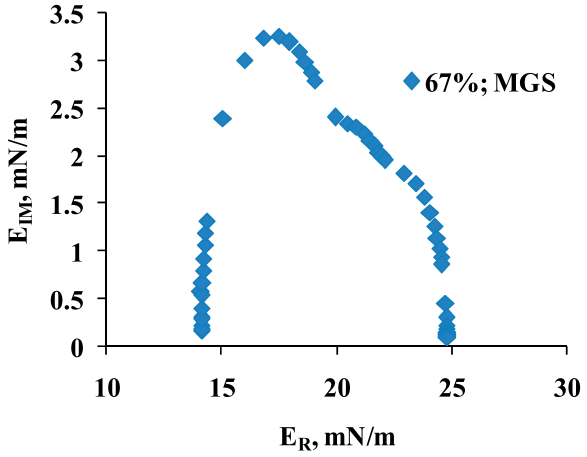

Supplement: Supplementary file 1 [file ijms-19-02209-s001.pdf]
